# Psychological Burden Among Informal Caregivers of Cirrhosis and Liver Transplant Patients: A Cross‐Sectional Study Using the Burden Scale for Family Caregivers

**DOI:** 10.1111/jep.70588

**Published:** 2026-09-08

**Authors:** Raquel Sanches Slusarski Martins, Luiz Augusto Carneiro D'Albuquerque, Gilton Marques Fonseca, Luciana Bertocco de Paiva Haddad

**Affiliations:** ^1^ Departamento de Gastroenterologia, Hospital das Clínicas HCFMUSP, Faculdade de Medicina Universidade de São Paulo São Paulo SP Brazil

**Keywords:** caregivers, chronic diseases, liver cirrhosis, liver transplant, psychological stress, psychosocial effects of the disease

## Abstract

**Objectives:**

To compare the burden and stress levels of two groups of informal caregivers: those assisting patients undergoing treatment for cirrhosis (Group 1) and those caring for liver transplant recipients (Group 2).

**Methods:**

This cross‐sectional study included 223 informal caregivers of cirrhosis and liver transplant patients monitored at the Liver Transplant Unit. Participants completed a sociodemographic questionnaire, a patient disease history form, and the Burden Scale for Family Caregivers (BSFC).

**Results:**

Caregiver burden was influenced by specific factors: being younger than 40 years, having children, caring for emotionally distressed patients, and lower per capita income and women were the most burdened.

**Conclusions:**

These findings highlight the need for public health strategies that recognize and support the essential, yet often invisible, role of informal caregivers. A more equitable redistribution of caregiving responsibilities between families, Society, and the State is critical to reducing caregiver strain and improving outcomes for both caregivers and patients.

## 1 Introduction

Chronic liver disease and cirrhosis represent major global public health challenges, accounting for approximately 2 million deaths annually, in addition to a substantial burden of disability and increased healthcare utilization. This burden has been driven primarily by the growing prevalence of metabolic dysfunction‐associated steatotic liver disease and alcohol‐related liver disease, whereas the burden attributable to viral hepatitis has declined as a result of expanded hepatitis B vaccination and the availability of direct‐acting antiviral therapies for hepatitis C [1].

Cirrhosis therefore remains a major cause of morbidity and mortality, with a higher prevalence among men and an increasing trend in hepatic decompensation and hepatocellular carcinoma, particularly among individuals with obesity, diabetes, and excessive alcohol consumption [1].

Globally, Sepanlou et al. [2] demonstrated that cirrhosis was responsible for approximately 1.32 million deaths and 41.4 million disability‐adjusted life years in 2017. Although the absolute number of cases increased between 1990 and 2017, age‐standardized mortality and disability‐adjusted life‐year rates declined, which may reflect advances in prevention strategies and the management of liver diseases. In 2017, deaths attributable to cirrhosis accounted for 2.4% (2.3–2.6) of all deaths worldwide, compared with 1.9% (1.8–2.0) in 1990.

Beyond the overall magnitude of the disease burden, these findings indicate a transition in the underlying causes of cirrhosis. The relative contribution of hepatitis B and hepatitis C declined, while the contribution of alcohol‐related liver disease and metabolic dysfunction‐associated steatohepatitis increased, particularly in high‐income countries [1, 2]. Nevertheless, the distribution of cirrhosis remains uneven across populations and geographic regions, with a greater burden observed among men and in regions such as Sub‐Saharan Africa and Central Asia [2].

Although liver transplantation can improve survival and reduce complications, it also introduces new challenges. Post‐transplant patients face ongoing risks such as immunosuppression‐related complications and diminished quality of life [3]. In this context, informal caregivers (family members, friends, neighbors, or anyone who provides primary care, even if they are not healthcare professional or have no training) play a fundamental role. They are responsible not only for physical assistance, such as medication management and to dietary restrictions, but also for providing emotional support and navigating healthcare logistics [4].

However, the caregiver's experience is often overlooked in clinical care and research, which traditionally focuses on the patient. Informal caregivers frequently endure high levels of psychological, physical, and financial stress [5, 6]. Many reduce work hours or leave employment entirely to meet caregiving demands, leading to financial hardship. These strains are exacerbated when the caregiver is also managing household responsibilities or childcare.

Gender disparities further compound caregiver burden. Women, particularly spouses, are more likely to assume caregiving roles due to persistent social expectations [7, 8]. This cultural dynamic places women at higher risk for chronic stress and emotional exhaustion.

Moreover, caregivers of patients with advanced liver disease often lack adequate training or support. Unpreparedness correlates with higher levels of anxiety and depression, and a lower perception of quality of life [8]. Tools like the Burden Scale for Family Caregivers (BSFC) enable structured evaluation of both objective and subjective aspects of caregiver strain, offering a pathway to targeted interventions [9].

Despite its critical relevance, the burden of caregiving in the context of cirrhosis and liver transplantation remains insufficiently studied (particularly in comparative analyses that consider both pre‐ and post‐transplant phases). This study aims to fill that gap by quantifying and comparing caregiver burden in these two clinical stages, while identifying demographic and psychosocial factors that influence stress levels.

## 2 Methods

### 2.1 Study Design—Empirical Research Quantitative

This cross‐sectional study included informal caregivers of patients with cirrhosis and liver transplant recipients followed at a tertiary‐level liver transplant center. The center performs approximately 140–160 liver transplantations annually, with 149 procedures performed in 2025. Only patients on the liver transplant waiting list remain under follow‐up at the center. The number of patients on the waiting list was 331 in 2022, 300 in 2023, 267 in 2024, and 296 in 2025.

Data collection was conducted during two distinct periods. In 2018 (Period 1), in the months of June, July, and August. And during the COVID‐19 pandemic, in 2021 (Period 2), in the months of August, September, October, November, and December. With a 3‐year interval between one data collection and the other.

### 2.2 Inclusion Criteria

Eligible participants were unpaid informal caregivers, aged 18 years or older, who had been providing care for at least 3 months to patients diagnosed with cirrhosis of any etiology or who had who underwent a liver transplant at least a week ago. Caregivers were required to be the primary providers of daily assistance, regardless of cohabitation status. All participants had to be capable of reading and signing the Informed Consent Form. Informal caregivers of liver re‐transplant patients were also included.

### 2.3 Exclusion Criteria

Caregivers were excluded if they were healthcare professionals or hired personnel, or if they had medical or psychiatric conditions that would impair participation. Additional exclusions included those who refused and did not sign the Informed Consent Form, those who did not complete the questionnaires, and caregivers of patients who had received combined organ transplants (e.g., liver‐heart).

### 2.4 Data Collection Procedures

Caregivers were approached during routine outpatient appointments at the Liver Transplant Unit. Following approval from the institutional Research Ethics Committee, participants were provided with a detailed explanation of the study objectives, procedures, and ethical considerations. After accepting and signing the Informed Consent Form, caregivers completed a demographic questionnaire, a patient clinical history form, and the Burden Scale for Family Caregivers (BSFC). The interviews were conducted individually and lasted approximately 30 min. Medical records were reviewed to supplement clinical data.

### 2.5 Burden Scale for Family Caregivers (BSFC)

The BSFC is a validated instrument developed by [9] to assess caregiver burden. It consists of 28 statements with four response options and includes both direct and reverse‐scored items to reduce response bias. The scale measures both subjective and objective burden in non‐professional caregivers assisting chronically ill patients at home. Previous studies report a standard deviation of approximately 18.7. A minimum score difference of 5, 10, or 15 points is considered clinically meaningful. Sample size estimation was based on these parameters, resulting in a target of 223 participants for 95% confidence and 5% margin of error [10, 11, 12].

### 2.6 Statistical Analysis

Quantitative variables were summarized using means, medians, standard deviations, and interquartile ranges. Categorical variables were presented as absolute and relative frequencies. Associations between categorical variables were analyzed using Pearson's chi‐square test or Fisher's exact test, as appropriate. The Mann–Whitney *U* test was used to compare two independent groups, and the Kruskal–Wallis test for comparisons among three or more groups. Logistic regression analyses (both univariate and multivariate) were conducted to identify independent predictors of caregiver burden. Backward stepwise elimination was applied to refine the final model. Statistical significance was set at *p* < 0.05. The analyses were performed using the software SPSS version 25.0 [13]. Occupations were categorized according to the International Standard Classification of Occupations [14].

### 2.7 Results

A total of 223 informal caregivers participated in the study, of which 120 were caring for patients with cirrhosis and 103 for liver transplant recipients. In Period 1, 10 informal caregivers declined participation; in Period 2, 14 declined and 23 did not return after being called for their patient's appointment and were excluded from the final analysis.

### 2.8 Characteristics of Patients With Cirrhosis and Liver Transplant Recipients

Patients with cirrhosis were significantly older at the time of diagnosis (mean age 47.8 years) compared to transplant recipients (mean age 39.1 years, *p* = 0.001)—Table 1. Several clinical markers showed statistically significant differences between the two groups, including total bilirubin, prothrombin time (PT), international normalized ratio (INR), serum albumin, serum sodium, and creatinine (all *p* < 0.001), reflecting physiological changes associated with the transplant process – Table 1.

**Table 1:** Clinical and demographic characteristics of patients with cirrhosis and liver transplant recipients followed at a Liver Transplant Unit.

Patient with cirrhosis		Patient with liver transplantation	
*n* (%) or M (SD)		*n* (%) or M (SD)	
Gender			*p‐*value
Male	80 (55.6%)	64 (44.4%)	
Female	40 (50.6%)	39 (49.4%)	0.481
Age at interview (in years)	53.9 (14.2%)	49,2 (16.9%)	0.089
Age at diagnosis (years)	47.8 (15.9%)	39.1 (19.1%)	**0.001**
Year of interview			
Period 1	53 (49.1%)	55 (50.9%)	0.169
Period 2	67 (58.3%)	48 (41.7%)	
Profession Group 3			
No	108 (51.4%)	102 (48.6%)	0.004
Yes	12 (92.3%)	1 (7.7%)	
Current occupation Group 3			
No	114 (52.5%)	103 (47.5%)	0.032
Yes	6 (100)	0	
Billirubin total mg/dL	2.69 (3.80)	0.56 (0,38)	**< 0.001**
TP	16.43 (5,03)	14.81 (11.66)	**< 0.001**
INR	1.35 (0.42)	1.13 (0.38)	**< 0.001**
Serum albumin g/dL	3.58 (0.78)	4.16 (0.67)	**< 0.001**
Na mEq/L	137.85 (4.65)	139.75 (2.46)	**< 0.001**
Serum creatinine mg/dL	1.02 (0.42)	1.25 (0.54)	**0.001**
MELD	14.5 (6.0)	17.3 (4.6)	0.261
Transplant list	53 (96.4)	2 (3.6)	**< 0.001**
Time on transplant list (months)	15.1 (27.1)	—	
Transplant time (months)	—	61.3 (67.7)	
Income (minimum wage)			
None	19 (48.7%)	20 (51.3%)	
Less than 1	33 (50.0%)	33 (50.0%)	0.629
1 to 3	42 (58.3%)	30 (41.7%)	
3 to 6	17 (47.2%)	19 (52.8%)	
More than 6	3 (75.0%)	1 (25.0%)	

*Note:* Pearson's chi‐square test; Fisher's exact test; Mann–Whitney test. Per capita income none: missing data = 6. Bolded values were intended to highlight the statistically significant differences in total bilirubin, PT, serum albumin, sodium (Na), and serum creatinine according to the performance of the transplatation procedure.

A higher proportion of liver transplant recipients had comorbidities or reported additional symptoms compared to cirrhotic patients (51.2% vs. 29.4%, *p* = 0.001 and *p* = 0.006, respectively). Prior surgical history was reported in 49.0% of cirrhotic patients and 51.0% of transplant recipients (*p* < 0.001). Viral hepatitis (B and C) was the most common etiology of cirrhosis in both groups.

### 2.9 Characteristics of Informal Caregivers of Patients With Cirrhosis and Liver Transplant Recipients

Caregivers of liver transplant recipients had been providing care for a longer period, with a mean duration of 111.3 months, compared to 68.3 months for caregivers of cirrhotic patients (*p* = 0.001). Women were the primary caregivers compared to men—Table 2. No significant differences were found in other sociodemographic variables between the two groups, except for time spent caregiving.

**Table 2:** Demographic and clinical characteristics of informal caregivers of cirrhosis patients and liver transplant recipients followed at a Liver Transplant Unit.

Caregivers of patients with cirrhosis		Caregivers of patients with liver transplant	
(*n*%) or *M* (SD)		(*n*%) or *M* (SD)	
Gender			*p*‐value
Male	18 (54.5%)	15 (45.5%)	
Female	102 (53.7%)	88 (46.3%)	0.927
Age at interview (in years)	47.9 (12.5%)	48.0 (12.7%)	0.866
Education Level			
Elementary incomplete	19 (52.8%)	17 (47.2%)	
Elementary	3 (23.1%)	10 (76.9%)	
Unfinished high school	3 (37.5%)	5 (62.5%)	0.018
High school	46 (51.1%)	44 (48.9%)	
Unfinished bachelor	8 (53.3%)	7 (46.7%)	
Bachelor	33 (62.3%)	20 (37.7%)	
Postgraduate education	8 (100%)	0	
Kinship to the care recipient			
Spouse	58 (48.7%)	57 (55.3%)	
Child	31 (26.1%)	16 (15.5%)	
Parents	15 (12,6%)	23 (22.3%)	
Sibling	9 (7.6%)	3 (2.9%)	
Grandmother	1 (0.8%)	0	
Grandson	1 (0.8%)	2 (1.9%)	
Cousin	0	1 (1.0%)	
Aunt	0	1 (1.0%)	
Daughter‐in‐law	1 (0.8%)	0	
Stepson	1 (0.8%)	0	
Ex‐wife/husband	1 (0.8%)	0	
Income (minimum wage)			
None	19 (48.7%)	20 (51.3%)	
Less than 1	33 (50.0%)	33 (50.0%)	0.059
1 to 3	42 (58.3%)	30 (41.7%)	
3 to 6	17 (47.2%)	19 (52.8%)	
More than 6	3 (75.0%)	1 (25.0%)	

*Note:* Pearson's chi‐square test; Fisher's exact test; Mann–Whitney Test.

### 2.10 Statistically Significant Association Between the Profession and Current Occupation of Patients With Cirrhosis and Liver Transplant Recipients in Relation to the Diagnosis

Significant differences were found in the occupational classification (Group 3: mid‐level technical and health professionals), with more cirrhosis patients belonging to this group than liver transplant recipients (*p* < 0.05). Similar results were distributed across the current occupation.

### 2.11 Impact of Emotional and Psychological Factors in Patients With Cirrhosis and Liver Transplant Recipients

Anxiety was more prevalent among liver transplant recipients (56.1%) than cirrhosis patients (43.9%). However, sadness was more commonly reported among cirrhotic patients (75.7%, *p* < 0.001). Liver transplant recipients were also more likely to receive psychological care (53.1% vs. 46.9%). The interview period and the patient's condition (diagnosis) were not factors associated with caregiver burden—Table 3.

**Table 3:** BSFC scores and sociodemographic characteristics of informal caregivers interviewed at a Liver Transplant Unit.

		Mean (SD)	Median (Q_1_–Q_3_ *)	*p*‐value
Cohort	period 1	29.64 (17.05)	28.50 (15.50–44.50)	0.738^a^
	period 2	28.85 (17.06)	27 (14–40)	
Patient's age (in years)	≤ 40 years old	27.3 (17.6)	21 (12–44)	0.562^b^
	41–50 years old	32.0 (16.5)	30 (44.5)	
	51–60 years old	30.3 (17.3)	28.9 (16.7)	
	> 60 years old	30 (16–40)	27 (14.5–42)	
Caregiver's age (in years)	≤ 40 years old	31.9 (17.6)	30 (18–49)	0.267^b^
	41–50 years old	27.7 (18.0)	24.5 (12–40)	
	51–60 years old	26.7 (15.8)	24 (15–38)	
	> 60 years old	31.0 (16.9)	31 (18–45)	
Caregiver's relationship with the patient**	Husband/Wife	30.3 (16.9)	31 (16–42.5)	0.940^a^
	Children	30.8 (16.5)	29.5 (18–41)	
Caregiver lives with the patient	No	25.86 (16.98)	24 (12–33)	0.155^a^
	Yes	30.02 (17.00)	30 (15.5–42,5)	

* Q1: first quartile; Q3: third quartile.

^a^ Mann–Whitney test.

^b^ Kruskal–Wallis test.

** Excluding other ties (siblings, parents, etc.) *n* = 158.

### 2.12 Caregiver Burden Analysis

**Figure 1:**
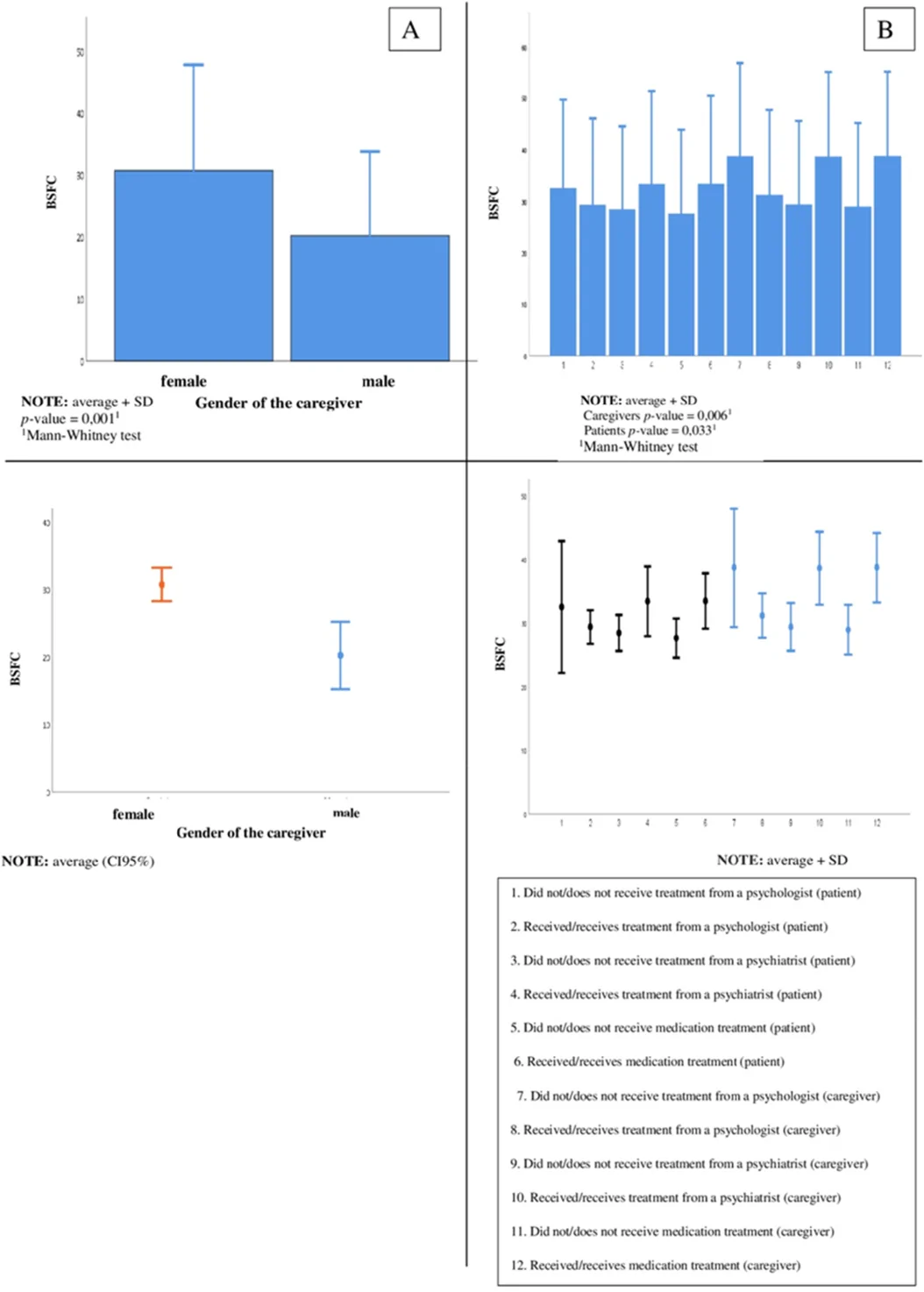
(A) BSFC according to the gender of the caregiver interviewed in a Liver Transplant Unit. (B) BSFC according to treatment with a psychologist, psychiatrist, and medications of caregivers and patients in the sample from the Liver Transplant Unit.

Caregiver gender was significantly associated with burden, with women reporting higher stress levels (see Figure 1A). Psychological distress in the patient particularly sadness was associated with increased caregiver burden (OR: 2.39, 95% CI: 1.11–5.14, *p* = 0.026). Additionally, caregiver burden was higher among those under 40 years old and among those with children (OR: 3.75, 95% CI: 1.38–10.21, *p* = 0.010)—Table 4.

**Table 4:** Factors associated with caregiver burden in cirrhosis and liver transplant settings: Results from logistic regression analysis.

		Unadjusted OR	Adjusted OR	
Variable		(CI95%)	(CI95%)	*p*‐value
Caregiver's age	≤ 40 years old	1	1	
	41–50 years old	0.70 (0.29–1.68)	0.41 (0.14–1.16)	0.092
	51–60 years old	0.50 (0.23–1.11)	0.27 (0.11–0.70)	0.007
	> 60 years old	1.07 (0.45–2.54)	0.93 (0.33–2.66)	0.893
Caregiver's per capita income	< 1 minimum wage	1	1	
	1–3 minimum wage	0.33 (0.16–0.71)	0.29 (0.12–0.70)	0.006
	> 3 minimum wage	0.26 (0.10–0.67)	0.18 (0.06–0.54)	0.002
Caregiver stress	No	1	1	
	Yes	1.92 (0.96–3.84)	2.19 (0.98–4.91)	0.056
Patient's sadness	No	1	1	
	Yes	3.38 (1.32–8.63)	2.39 (1.11–5.14)	0.026
Does the caregiver have children?	No	1	1	
	Yes	2.55 (1.07–6.09)	3.75 (1.38–10.21)	0.010
Group	period 1	1	1	
	Period 2	0.73 (0.40–1.35)	0.64 (0.31–1.31)	0.223
Diagnosis of the patient	Cirrhosis	1	1	
	Liver transplantation	1.01 (0.55–1.85)	1.16 (0.55–2.44)	0.699

Income was a protective factor: caregivers with 1–3 minimum wages had a 71% lower chance of experiencing burden (*p* = 0.006), and those earning more than 3 minimum wages had an 82% lower chance (*p* = 0.002). No significant associations were found between caregiver burden and marital status, COVID‐19 history, or whether the caregiver cohabitated with the patient—Table 4.

## 3 Discussion

This study contributes original insights into the psychosocial burden experienced by informal caregivers of patients with cirrhosis and liver transplant recipients. By applying the internationally validated Burden Scale for Family Caregivers (BSFC), the findings provide a robust foundation for designing targeted interventions to relief caregiver stress and ultimately benefiting both patients and healthcare systems.

The absence of significant differences in burden between caregivers of cirrhotic patients and those caring for liver transplant recipientes, suggests that the conditions under which care is provided, rather than the specific stage of the disease, may determine stress levels. Similarly, no significant differences were found between time periods, indicating that the duration of caregiving alone may not be the primary determinant of overload, although prolonged caregiving is known to contribute to physical and emotional fatigue [15].

Age emerged as a relevant fator, caregivers aged 40 or younger presented higher stress levels, likely reflecting the additional demands of managing professional, familial, and caregiving responsibilities simultaneously. These younger caregivers may also experience greater social isolation and disrupted family dynamics. On the other hand, caregivers aged 51–60 years presented lower burden, possibly due to accumulated experience, availability of social support, or retirement status reducing role conflict [15, 16].

Health issues were common among caregivers, but self‐care was frequently neglected. Cardiovascular symptoms predominated among those caring for cirrhosis patients, while endocrine disorders were more common among caregivers of liver transplant recipientes. Despite the health problems reported, many caregivers do not seek medical care for themselves. Even after transplantation, the patient remains the primary focus of care due to other ongoing health conditions and symptoms, such as memory lapses, diabetes, weight gain, and renal insufficiency, as reported by participants in our study. Consequently, caregivers may remain engaged in caregiving for prolonged periods [16, 17].

Nutritional management emerged as a particularly burdensome domain, given the complexity of dietary restrictions associated with cirrhosis, liver trasnplantation, diabetes, and renal dysfunction. Both, patients and informal caregivers struggled to adhere to these regimens, leading to frustration and increased emotional strain [18, 19]. Educational interventions and practical tools aimed at simplifying nutritional guidance may offer significant relief.

The emotional state of patients influenced caregiver burden. Caring for individuals experiencing sadness or anxiety was associated with significantly higher stress levels among caregivers, reinforcing the importance of dyadic interventions that address the psychological well‐being of both the patient and the caregiver [20].

Symptoms such as ascites, hepatic encephalopathy, and cognitive fluctuations may lead to hospitalization or require more frequent medical consultations, potentially preventing patients with cirrhosis from maintaining employment and resulting in substantial financial consequences. These economic impacts may be further exacerbated by the need for a more restrictive and costly diet, as well as expenses related to medications and wound care supplies [21, 22].

Moreover, even when pharmacological treatment is effective, the likelihood of returning to work may remain limited depending on the patient's clinical characteristics [21, 23].

Consequently, the caregiver may become the primary income provider for the household. In this context, disruption of employment, restrictions on family and social life, increased healthcare‐related expenses, and reduced household income may contribute to greater emotional distress among patients with cirrhosis, as observed in the sample of the present study.

These findings underscore the importance of ensuring that care is not limited to the transplantation process, including both the pre‐ and post‐transplant periods, and highlight the essential role of a multidisciplinary healthcare team. This team plays a fundamental role in providing guidance and support regarding the risks associated with these conditions, psychopharmacological treatments, individual and group therapies, and available support services [21, 24].

Understanding how patients and their caregivers cope with the disease is fundamental to the progression and effective management of the care process.

The perception that healthcare professionals are genuinely concerned about both patients and their caregivers, providing a supportive environment in which questions and difficulties can be openly addressed, while communicating information in an accessible manner and seeking to understand their individual experiences, may promote greater awareness, trust, and confidence in the care provided. Such support extends beyond healthcare settings and may contribute to the patients' and caregivers' ability to cope with the challenges associated with the disease in their daily lives [21, 24].

In contrast, continued support beyond the transplant evaluation process, including psychological support, is essential, given the challenges that persist. As evidenced by the patients in our sample, who exhibited high levels of anxiety and continued to receive psychological counseling and pharmacological treatment for mental health conditions, these challenges may significantly affect post‐transplant adjustment. Following the procedure, patients may experience difficulties returning to work due to fatigue and frequent medical appointments, as well as restrictions and changes in lifestyle, including dietary, social, and physical activities [15, 25, 26]. Furthermore, our findings indicate that the mean age of liver transplant recipients was 49.2 years and that an income at or below the minimum wage may also contribute to difficulties in returning to work and to the perception that there has been no improvement in quality of life.

This is further reinforced by reports that some patients fail to adhere to the medication regimen required to prevent organ rejection, while others may attempt suicide, as reported by caregivers in the study. Consequently, caregivers may assume responsibility for monitoring and administering medications [15, 25]. Thus, our findings are relevant in highlighting the relationship between liver transplantation, anxiety, and emotional distress, with important implications for both patients and caregivers [27].

Interestingly, the COVID‐19 pandemic did not significantly exacerbate caregiver burden in this sample. This may be attributable to the quality of outpatient follow‐up at the study center, which helped maintain continuity of care despite external disruptions.

The caregiver has a great deal of information about the patient. However, the omission of data such as income or failure to return to complete the questionnaire was one of the limitations of this study, hindering the ability to understand the patient's needs and offer appropriate support and guidance.

Socioeconomic status was a strong predictor of caregiver burden. Lower per capita income was associated with greater stress, likely reflecting the financial strain of reduced work hours, job loss, or the costs of long‐term care. These findings underscore the need for policies that provide economic and logistical support to informal caregivers, particularly those in financially vulnerable positions [15].

Our study indicates that women are still predominantly associated with the role of instinctive caregiving, which limits the possibility of receiving financial compensation for the demanding process of providing care, particularly to patients with chronic illnesses. This responsibility disproportionately affects healthy wives and daughters, with the caregiving role being passed down from one generation to the next.

In addition, caregivers who also had children presented significantly higher stress levels, reflecting the compounding responsibilities of intergenerational caregiving. This aligns with evidence that caregiving remains a gendered and invisible form of labor, often assumed by women without institutional support [7, 21].

Involving healthcare professionals in caregiver education and emotional support can help reduce anxiety and increase confidence in care delivery. Expanded outpatient services, home‐based care, and structured mental health programs for caregivers may prevent long‐term deterioration and burnout. Future longitudinal studies are needed to clarify causality and track the evolution of caregiver burden over time.

## 4 Conclusion

Care is a fundamental human activity that intersects with social, economic, health, and educational domains. This study underscores the urgent need for a more balanced redistribution of caregiving responsibilities between families, Society, and the State. Recognizing informal caregiving as legitimate labor is a critical step toward addressing caregiver burden. Public policies that support caregivers, through financial assistance, flexible work arrangements, access to mental health care, and educational resources, are essential. Such initiatives would not only relief psychological stress among caregivers but also improve patient outcomes and reduce long‐term healthcare costs.

## Ethics Statement

This study received ethics approval from Ethics Committee for Analysis of Research Projects (CAPPesq) of Hospital das Clínicas HCFMUSP, Faculdade de Medicina, Universidade de São Paulo, under opinion number 2,576,080 dated April 3, 2018 and under opinion number 4,565,102 dated March 1, 2021.

## Consent

All caregivers and patients provided consent for participation in the study by signing the Informed Consent Form (ICF).

## Conflicts of Interest

The authors declare no conflicts of interest.

## Artificial Intelligence Use

In addition to a professional proofreader, an artificial intelligence tool (ChatGPT) was used exclusively to review the English and avoid spelling and interpretation erros in the text.

## Data Availability

The data supporting the findings of this study are included in the manuscript.
